# Pathophysiology of NSAID-Associated Intestinal Lesions in the Rat: Luminal Bacteria and Mucosal Inflammation as Targets for Prevention

**DOI:** 10.3389/fphar.2018.01340

**Published:** 2018-11-29

**Authors:** Rocchina Colucci, Carolina Pellegrini, Matteo Fornai, Erika Tirotta, Luca Antonioli, Cecilia Renzulli, Emilia Ghelardi, Elena Piccoli, Daniela Gentile, Laura Benvenuti, Gianfranco Natale, Federica Fulceri, Pablo Palazón-Riquelme, Gloria López-Castejón, Corrado Blandizzi, Carmelo Scarpignato

**Affiliations:** ^1^Department of Pharmaceutical and Pharmacological Sciences, University of Padua, Padua, Italy; ^2^Department of Clinical and Experimental Medicine, University of Pisa, Pisa, Italy; ^3^Reasearch & Development Department, Alfasigma SpA, Bologna, Italy; ^4^Department of Translational Research and New Technologies in Medicine and Surgery, University of Pisa, Pisa, Italy; ^5^Manchester Collaborative Centre for Inflammation Research, University of Manchester, Manchester, United Kingdom; ^6^Clinical Pharmacology & Digestive Pathophysiology Unit, Department of Clinical & Experimental Medicine, University of Parma, Parma, Italy

**Keywords:** non-steroidal anti-inflammatory drugs, intestinal damage, intestinal bleeding, rifaximin, enteroprotection, microbiota

## Abstract

Non-steroidal anti-inflammatory drugs (NSAIDs) can damage the small intestine, mainly through an involvement of enteric bacteria. This study examined the pathophysiology of NSAID-associated intestinal lesions in a rat model of diclofenac-enteropathy and evaluated the effect of rifaximin on small bowel damage. Enteropathy was induced in 40-week old male rats by intragastric diclofenac (4 mg/kg BID, 14 days). Rifaximin (delayed release formulation) was administered (50 mg/kg BID) 1 h before the NSAID. At the end of treatments, parameters dealing with ileal damage, inflammation, barrier integrity, microbiota composition, and TLR-NF-κB-inflammasome pathway were evaluated. In addition, the modulating effect of rifaximin on NLRP3 inflammasome was tested in an *in vitro* cell system. Diclofenac induced intestinal damage and inflammation, triggering an increase in tissue concentrations of tumor necrosis factor and interleukin-1β, higher expression of TLR-2 and TLR-4, MyD88, NF-κB and activation of caspase-1. In addition, the NSAID decreased ileal occludin expression and provoked a shift of bacterial phyla toward an increase in *Proteobacteria* and *Bacteroidetes* abundance. All these changes were counterbalanced by rifaximin co-administration. This drug was also capable of increasing the proportion of Lactobacilli, a genus depleted by the NSAID. In LPS-primed THP-1 cells stimulated by nigericin (a model to study the NLRP3 inflammasome), rifaximin reduced IL-1β production in a concentration-dependent fashion, this effect being associated with inhibition of the up-stream caspase-1 activation. In conclusion, diclofenac induced ileal mucosal lesions, driving inflammatory pathways and microbiota changes. In conclusion, rifaximin prevents diclofenac-induced enteropathy through both anti-bacterial and anti-inflammatory activities.

## Introduction

Non-steroidal anti-inflammatory drugs (NSAIDs) represent one of the most widely used classes of drugs (McGettigan and Henry, 2013). Despite they confer important clinical benefits across multiple indications, their use is associated with a wide array of adverse events involving different organs and systems (i.e., liver, kidney, cardiovascular system, skin, and gut). Those concerning the gastrointestinal tract are the most common and can cause both symptoms (dyspepsia, heartburn, and/or abdominal discomfort) and lesions (mucosal erosions and/or peptic ulcers, with their life-threatening ulcer complications of bleeding and perforation) (Lanas and Hunt, 2006; Scarpignato and Hunt, 2010). While NSAID-induced renal adverse events are well-known, those concerning cardiovascular system (Grosser et al., 2017; Patrono and Baigent, 2017) have been appreciated only recently, after the rofecoxib fallout (DeMaria, 2004). Several investigations (like the LOGICA and the GIRANO studies) have shown that patients requiring NSAID therapy often display gastrointestinal or/and cardiovascular risk factors (Lanas et al., 2010; Vanderstraeten et al., 2016). Navigating through the different risk factors and balancing them with the potential benefits of NSAID therapy is clearly a difficult task.

Since in real life NSAID-associated gastrointestinal adverse events largely exceed the cardiovascular ones (Lanas et al., 2015), these drugs are frequently co-prescribed with proton pump inhibitors (PPIs) to prevent NSAID-associated gastro-duodenal damage and symptoms. According to currently available guidelines (Tielemans et al., 2010; Scarpignato et al., 2015) the co-administration of a gastroprotective compound (especially a PPI) in NSAID users, holding one or more gastrointestinal risk factors, has become the standard of care. Indeed, acid represents the *condition sine qua non* for development of peptic ulcer and related complications (Scarpignato and Pelosini, 1999). However, since NSAID-enteropathy is not a pH-dependent phenomenon, PPIs do not exert any protective activity beyond the duodenum (Scarpignato et al., 2015).

Already, in the early 80s, some studies (Bjarnason et al., 1984, 1987) showed that NSAID-associated gastrointestinal damage does extend also to the lower digestive tract. Later on, the clinical impact of lower gastrointestinal damage and complications was pointed out mainly by the first large prevention study with the synthetic prostaglandin, misoprostol (i.e., the MUCOSA trial) (Silverstein et al., 1995). In addition, the VIGOR trial (comparing rofecoxib with naproxen) reported that more than 40% of NSAID-related events occurred in the lower gastrointestinal tract (Laine et al., 2003). Over the past decade, the overall pattern of gastrointestinal events requiring hospitalization showed a reverse trend, with a decrease of events located in the upper gastrointestinal tract and a slight, albeit significant, increase in those concerning the small and large bowel (Lanas et al., 2009).

The advent of new imaging techniques, such as video capsule endoscopy, has allowed to gain new insights into NSAID-induced intestinal damage, which appears to be site-dependent. The pattern of mucosal injury, which can be found in up to 75% of NSAID users (Graham et al., 2005; Maiden et al., 2005), ranges from denuded areas (seen mainly in the proximal small bowel) to the so-called mucosal breaks (erosions and ulcers), observed in its distal part (Fujimori et al., 2010). Video capsule studies and/or fecal calprotectin measurement clearly showed that PPIs (like omeprazole) failed to prevent NSAID-associated intestinal injury both in healthy volunteers (Goldstein et al., 2005, 2007; Maiden et al., 2005) and patients (Graham et al., 2005). Recent experimental (for review see Blackler et al., 2014) and clinical (Fujimori et al., 2010; Washio et al., 2016) evidence suggest that PPIs may actually aggravate NSAID injury in the small bowel.

The pathogenesis of small intestinal damage is still not completely understood. The synthesis of endogenous mucosal prostaglandins is inhibited by NSAID throughout the *entire* gastrointestinal tract. However, other important pathogenic factors, which contribute to damage, differ between the distal and proximal regions of the gut (Bjarnason et al., 2018). Indeed, in the small bowel, the presence of bacteria and bile represent pivotal triggers of the mucosal damage (Lanas and Scarpignato, 2006; Scarpignato, 2008; Blackler et al., 2014).

Bile acids (Petruzzelli et al., 2007) and inhibition of endogenous mucosal prostanoids (Takeuchi et al., 2010) are both implicated in the pathogenesis of NSAID-enteropathy. However, current interest is focused on NSAID-induced alterations in the gut microbiota, with consequent pathological activation of the downstream inflammatory pathways (Scarpignato, 2008; Bjarnason et al., 2018). Recent evidence (for review see Pellegrini et al., 2017) points out the importance of nucleotide-binding oligomerization domain leucine rich repeat and pyrin domain-containing protein 3 (NLRP3) inflammasome in the interaction amongst gut microbiome, intestinal barrier and innate immune system, controlling in this way intestinal homeostasis. A recent study (Higashimori et al., 2016) suggested that NLRP3 inflammasome activation and associated IL-1β release play a key role in NSAID-induced enteropathy.

The pathogenic role of enteric bacteria is supported by a large experimental evidence (for review see Lanas and Scarpignato, 2006; Scarpignato, 2008; Blackler et al., 2014) showing that anti-microbials attenuate NSAID-enteropathy. In humans (Bjarnason et al., 1992), metronidazole (an anti-microbial targeting most Gram-negative and Gram-positive anaerobic bacteria, Freeman et al., 1997) was shown to reduce inflammation and blood loss in NSAID users, thus suggesting a therapeutic role for anti-microbials in this clinical setting. However, potential adverse effects as well as the risk of drug resistance, associated with the use of systemic anti-microbials, have so far precluded this interesting approach (Lanas and Scarpignato, 2006).

Rifaximin (4-deoxy-4′-methylpyrido[1′,2′-1,2]imidazo [5,4-c]rifamycin SV) is a semi-synthetic rifamycin derivative, retaining the broad spectrum of anti-bacterial activity shared by all the members of this drug class, but displaying a very low (<1%) absorption from the gastrointestinal tract (Calanni et al., 2014). Being poorly absorbed, it achieves intraluminal concentrations much higher than those needed to inhibit enteric bacteria. In addition, since it does not reach the systemic circulation, the risk of anti-microbial resistance of adverse effects is rather low (Scarpignato and Pelosini, 2005). Originally approved for the treatment of gastrointestinal infections, the use of rifaximin has been subsequently extended to other organic (like diverticular disease and hepatic encephalopathy) and functional (such as irritable bowel syndrome and functional constipation) diseases of the digestive system (Scarpignato and Pelosini, 2005, 2006). Recently, the drug has been studied also in patients with inflammatory bowel disease (Gionchetti et al., 2006; Prantera et al., 2012).

Since rifaximin displays all the characteristics of an ideal antibiotic for targeting enterobacteria (DuPont and Ericsson, 1993), its ability to prevent NSAID-induced intestinal damage has been evaluated in both experimental (Ciobanu et al., 2014; Fornai et al., 2016) and clinical (Scarpignato et al., 2017) studies. In healthy volunteers indeed the number and severity of diclofenac-induced intestinal lesions (as evaluated by video capsule endoscopy) were reduced by co-administration of the drug (Scarpignato et al., 2017).

To gain deep insights into the pathophysiology of NSAID-associated lesions and to unravel the mechanisms of rifaximin enteroprotective activity, we employed an animal model of diclofenac-enteropathy and studied the effects of this poorly absorbed antibiotic on mucosal lesions, inflammatory changes and underlying molecular patterns as well as alterations of gut microbiota induced by the NSAID. For this investigation, a recently developed formulation (designed to bypass the stomach and release the microgranules directly in the intestine) (Prantera et al., 2012) was selected. It consists of microgranules of rifaximin, coated with a gastric acid-resistant polymer, which increases the intraluminal concentration of the drug, thus maximizing its therapeutic efficacy.

## Materials and Methods

### Study Design

The present study includes experiments based on morphological (histological evaluations), molecular (biochemical and protein expression assays), and microbiological (analysis of intestinal microbiota composition) techniques to examine the protective effects of rifaximin against NSAID-induced enteropathy in rats. Groups of animals were randomly assigned to treatments with drug vehicle, diclofenac, rifaximin or their combination. In all experiments, investigators performing laboratory analyses (which were made in duplicate) were blinded to the treatment protocol. The sample size (*n* = 10 per group) was based on the resource equation because the effects size was unknown (Charan and Kantharia, 2013). Numbers of animals for each experiment are detailed below and included in the figure legends.

### Animals

Experiments were performed on aged (40-week old) male Wistar rats (500–600 g). The animals were fed standard laboratory chow (Envigo, Udine, Italy) and tap water *ad libitum*, and were not employed for at least 1 week after their delivery to the laboratory. They were housed in solid-bottomed cages, equipped wire-mesh bottom inserts to prevent coprophagy and located in temperature-controlled rooms (at 22–24°C and 50–60% humidity) under a 12-h light cycle (06:00–18:00 h). Their care and handling were in accordance with the provisions of the European Community Council Directive 210/63/UE, recognized and adopted by the Italian Government. The experiments were approved by the Ethical Committee for Animal Experimentation of the University of Pisa and by the Italian Ministry of Health. The *in vivo* studies are reported in accordance with the ARRIVE guidelines for reporting experiments involving animals (Kilkenny et al., 2010).

### Experimental Design

Enteropathy was induced by diclofenac in accordance with the method previously developed in our laboratory (Fornai et al., 2014). The dose of diclofenac was selected on the basis of our previous study, in which preliminary experiments were conducted to identify the timing and dose required to elicit lower gastrointestinal injury. In that study, the effects of increasing doses of diclofenac (2, 4, and 6 mg/kg), administered by gavage twice daily, were assessed at days 7 and 14 of treatment (Fornai et al., 2014). The results showed that diclofenac, administered at 4 mg/kg BID, was able to produce the development of small intestinal damage after 14 days – consistently with human data (Scarpignato et al., 2017) – without causing excessive mortality and, at the same time, remaining within the range of its inhibitory activity for both COX isoenzymes.

Rifaximin (suspended in 1% methylcellulose, 1 ml/rat) was administered as an extended intestinal release (E.I.R.) formulation (50 mg/kg BID) 1 h before diclofenac for 14 days. Due to the high frequency of treatments, intragastric administration of test drugs was performed in awake animals. Twenty-four hours after the last dose of test drugs, non-fasted rats were anesthetized with chloral hydrate. Blood samples were collected by cardiac puncture from each animal for hemoglobin measurement. Fecal pellets were collected directly from the sigmoid colon and stored at -80°C for calprotectin measurement. The whole gastrointestinal tract was excised and examined macroscopically. Samples of ileum were snap frozen in liquid nitrogen and stored at -80°C for subsequent analysis of myeloperoxidase (MPO), malondialdehyde (MDA), tumor necrosis factor (TNF) and interleukin-1β (IL-1β), as well as to assess: the expression of toll-like receptors-2 and -4 (TLR-2, TLR-4), and the myeloid-differentiation primary response gene 88 (MyD88), nuclear factor-κB (NF-κB) signaling and activation of caspase-1, and the expression of the tight junction protein occludin. Ileal samples were also processed for the evaluation of the main bacterial phyla abundance as well as the prevalence of the genus *Lactobacillus*, as reported below. Other portions of ileal tissue were fixed in 10% formalin for subsequent evaluation of microscopic damage.

The experimental groups were arranged as follows: **Group 1**: animals treated with vehicle (control, *n* = 10); **Group 2**: animals treated with diclofenac (*n* = 15); **Group 3**: animals treated with diclofenac plus rifaximin (*n* = 12); **Group 4**: animals treated with rifaximin alone (*n* = 12). Different numbers of animals for each group of treatment were employed to compensate for the different mortality rates affecting the groups of treatment (as observed also in our previous studies, Fornai et al., 2014), in order to ensure at least 10 animals per group at the end of treatments.

### Assessment of Intestinal Damage, Tissue Inflammation, and Tissue Cytokine Concentrations

Histology of the small bowel injury, tissue myeloperoxidase (MPO, as an index of intestinal wall infiltration of polymorphonuclear cells) and malondialdehyde (MDA, reflecting membrane lipid peroxidation) concentrations were measured according to previously adopted methods (Fornai et al., 2016), detailed in Supplementary Material.

Tumor necrosis factor and Interleukin-1β (IL-1β) ileal concentrations were quantitated according to the methodology adopted in our laboratories (Fornai et al., 2016; Pellegrini et al., 2016). Tissue preparation is described in Supplementary Material.

### Measurement of Fecal Calprotectin and Blood Hemoglobin Concentration

Calprotectin, a calcium binding protein of neutrophil granulocytes that correlates well with neutrophil infiltration of the intestinal mucosa, was measured in fecal pellets, collected as reported above whereas hemoglobin analysis was performed on blood samples as previously described (Fornai et al., 2016) (see Supplementary Material).

### Western Blot Analyses

Specimens of mucosa were excised from ileum, weighed and homogenized in lysis buffer, using a polytron homogenizer. Mucosal homogenates were spun by centrifugation at 15,000 *g* for 15 min at 4°C, and the resulting supernatants were then separated from pellets and stored at -80°C for subsequent quantification of TLR-2, TLR-4, MyD88, NF-κB p65 subunit, caspase-1 and occludin expression. The detailed procedure as well as primary and secondary antibodies employed are described in Supplementary Material and in the Supplementary Tables S1, S2.

### Analysis of Bacterial Populations in Ileal Samples

Total DNA extracted from ileal tissues was analyzed for the mucosal-associated microbiota through 16S rDNA metagenomics. Metagenomic analysis was performed with MiSeq, Illumina platform at GenProbio SRL (Parma, Italy). The methodology employed to extract DNA from tissues as well the detailed procedure of 16S RNA gene amplification and MiSeq sequencing are detailed in Supplementary Material.

### *In vitro* Assays on NLRP3 Inflammasome

The human monocyte cell line THP-1, a well-established model to study monocyte/macrophage functions, mechanisms, and signaling pathways (Chanput et al., 2014), were cultured in RPMI 1640 media, supplemented with 10% fetal bovine serum, 100 units/ml penicillin, and 100 μg/ml streptomycin. Cells were plated in 24-well plates at a density of 5 × 10^5^ cells/well and treated with phorbol 12-myristate 13-acetate (0.5 μM). After 3 h, the medium was removed, fresh medium was added, and cells were incubated overnight (37°C, 5% CO_2_). Cells were primed with lipopolysaccharide (1 μg/ml, 4 h) to induce pro-IL-1β expression before treatment with nigericin (a standard NLRP3 inflammasome activator (10 μM, 1 h) (Mariathasan et al., 2006), as described by Lopez-Castejon et al. (2013). Lipopolysaccharide-primed cells were treated for 2 h with rifaximin (25, 50, or 100 μM) before the addition of nigericin. Thereafter, lipopolysaccharide-primed and nigericin-treated cells (in the presence or absence of rifaximin) were incubated for a total of 5 h before the collection of supernatants and cell lysates for western blot assay of caspase-1 and measurement of IL-1β concentrations, as described in Supplementary Material.

### Statistical Analysis

Results are presented as mean ± standard error of mean (SEM). The statistical significance of data was evaluated by one-way analysis of variance (ANOVA) followed by *post hoc* analysis by Student–Newman–Keuls test, and *p*-values less than 0.05 were considered significant. All statistical calculations were performed using GraphPad Prism^TM^ 3.0 software (GraphPad, San Diego, CA, United States).

## Results

### Macroscopic Appearance of the Intestine

A quantitative evaluation of the macroscopic injury elicited by diclofenac was difficult to perform, due to the large mucosal surface. However, diaphragm-like strictures and multiple ulcerative lesions, both hallmark of NSAID-enteropathy, were clearly present in rats given the NSAID, a picture not seen in animals treated with diclofenac plus rifaximin or rifaximin alone. Representative images, showing the macroscopic appearance of small intestine in the different treatment groups, are displayed in Supplementary Figure 1.

### Microscopic Assessment of Intestinal Damage

**FIGURE 1:**
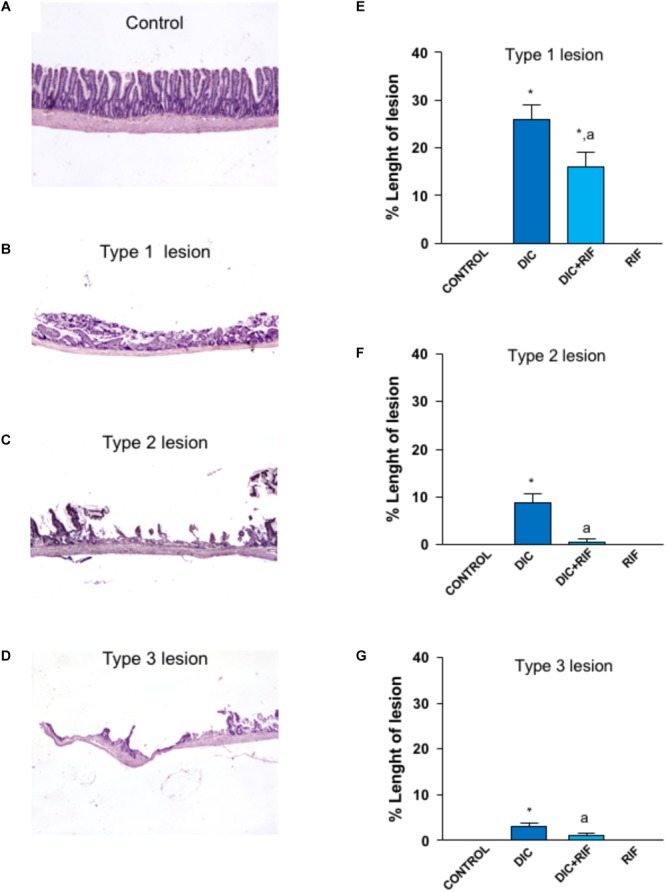
Histological analysis of damage in the ileum of rats treated with vehicle (Control), diclofenac (DIC, 4 mg/kg BID), diclofenac plus rifaximin (DIC+RIF, 50 mg/kg BID), or rifaximin (RIF) for 14 days. Representative pictures showing the microscopic appearance of ileal mucosa from control animals **(A)**, as well as type 1 **(B)**, type 2 **(C)** or type 3 **(D)** lesions, observed in animals treated with DIC. Effects of treatments on type 1 **(E)**, type 2 **(F)**, or type 3 **(G)** lesions. Each column represents the mean ± SEM obtained from 10 animals. ^∗^*p* < 0.05, significant difference versus Control; ^a^*p* < 0.05, significant difference vs. diclofenac alone.

In the ileum from control animals, microscopic examination did not reveal any type of lesion as was the case for rats treated with rifaximin alone. Administration of diclofenac was associated with the appearance of types 1, 2, and 3 lesions in the ileum (Figure 1). In animals treated with diclofenac+rifaximin, all types of NSAID-induced lesions were significantly decreased, as compared with rats treated with diclofenac alone (Figure 1).

### Hemoglobin Blood Levels

**FIGURE 2:**
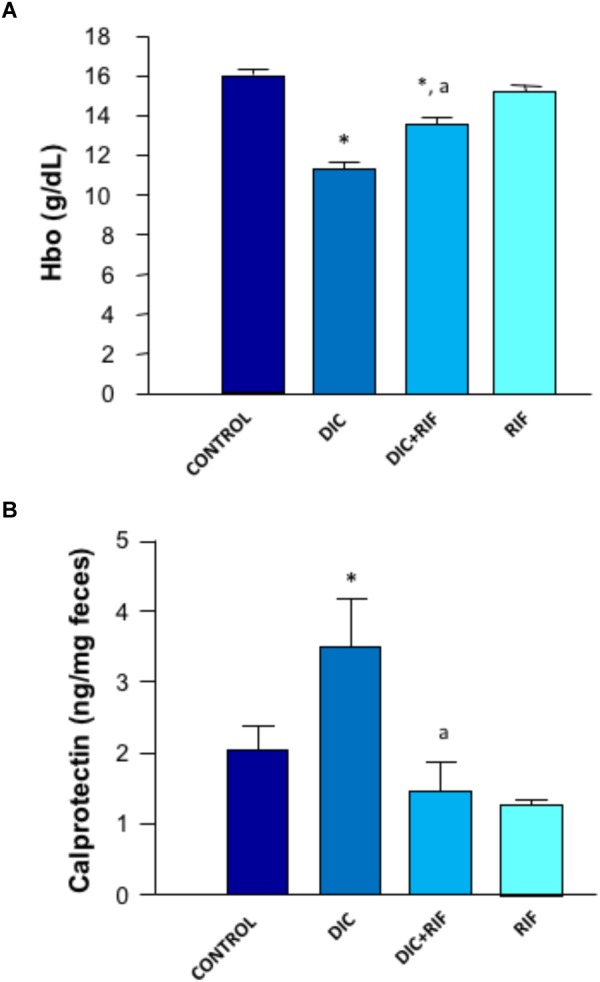
Effects of vehicle (Control), diclofenac (DIC, 4 mg/kg BID), diclofenac plus rifaximin (DIC+RIF, 50 mg/kg BID), or rifaximin (RIF) for 14 days on blood hemoglobin **(A)** or fecal calprotectin levels **(B)**. Each column represents the mean ± SEM obtained from 10 animals. ^∗^*p* < 0.05, significant difference versus Control; ^a^*p* < 0.05, significant difference versus diclofenac alone.

Mean hemoglobin concentration in the blood of control rats was 16.0 g/dL. A similar figure was obtained in animals given rifaximin alone. Compared to controls, diclofenac treatment induced a significant blood loss, as mirrored by the drop of blood hemoglobin concentration. This decrease was blunted by co-administration of rifaximin (Figure 2A). However, the hemoglobin levels of animals treated with both diclofenac and rifaximin did not achieve those of control animals, since upper gastrointestinal bleeding was likely not prevented by the antibiotic.

### Fecal Calprotectin Levels

The concentration of calprotectin in feces collected from control animals was 2.03 ng/mg feces. Animals treated with diclofenac displayed higher concentrations of fecal calprotectin (3.48 ng/mg feces) (Figure 2B). In rats treated with diclofenac+rifaximin, the levels of calprotectin were significantly reduced, approaching control values. Animals treated with rifaximin alone displayed levels of fecal calprotectin not significantly different from those observed in control animals.

### Tissue MPO Levels

**FIGURE 3:**
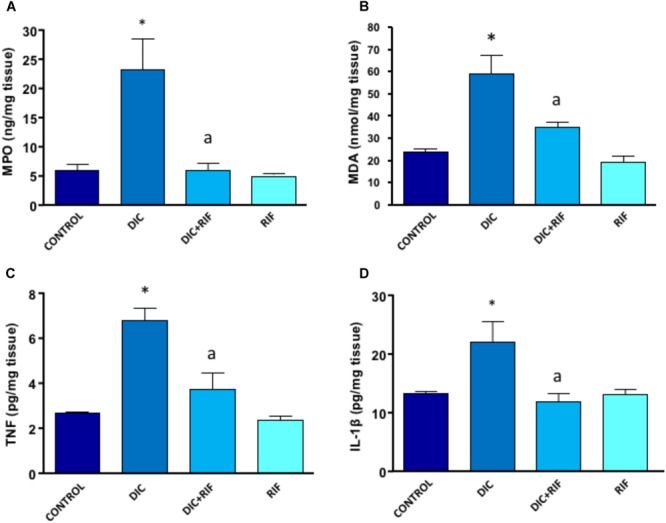
Effects of vehicle (Control), diclofenac (DIC, 4 mg/kg BID), diclofenac plus rifaximin (DIC+RIF, 50 mg/kg BID), or rifaximin (RIF) for 14 days on myeloperoxidase (MPO) **(A)**, malondialdehyde (MDA) **(B)**, tumor necrosis factor (TNF) **(C)**, or interleukin-1β (IL-1β) **(D)** in the ileum. Each column represents the mean ± SEM obtained from 10 animals. ^∗^*p* < 0.05, significant difference versus Control; ^a^*p* < 0.05, significant difference versus diclofenac alone.

Myeloperoxidase levels in ileal specimens taken from control rats accounted for 5.98 ng/mg tissue (Figure 3A) and were not modified by rifaximin alone. In animals treated with diclofenac, MPO levels were significantly increased (23.57 ng/mg tissue) (Figure 3A). In the ileum of rats treated with diclofenac+rifaximin, MPO levels were lower as compared with diclofenac alone and reverted back to the values of control animals.

### Tissue MDA Levels

In the ileum of control rats, the MDA concentration was 24.06 nmol/mg of tissue and, again, was not changed by administration of rifaximin. Animals treated with diclofenac displayed a significant increment of ileal MDA levels (59.1 nmol/mg of tissue) (Figure 3B), which were significantly reduced by co-administration of rifaximin.

### Tissue TNF Levels

TNF levels in ileum from control rats accounted for 2.65 pg/mg of tissue, and were not modified by treatment with rifaximin alone. The administration of diclofenac was associated with a significant increase in TNF concentration (Figure 3C). Compared with rats given diclofenac alone, animals treated with diclofenac+rifaximin showed a significant decrease in tissue TNF concentrations. However, the observed values were still higher (albeit not significantly different) than those observed in control animals (Figure 3C).

### Tissue IL-1β Levels

In control rats, IL-1β levels accounted for 13.2 pg/mg tissue and were not affected by treatment with rifaximin alone (Figure 3D). Rats treated with diclofenac displayed a significant increase in tissue IL-1β levels, as compared with control animals. Under these conditions, the concomitant administration of rifaximin was associated with a normalization of ileal levels of the cytokine (Figure 3D).

### Expression of TLR-2, TLR-4, MyD88, NF-κB p65 Subunit and Caspase-1

**FIGURE 4:**
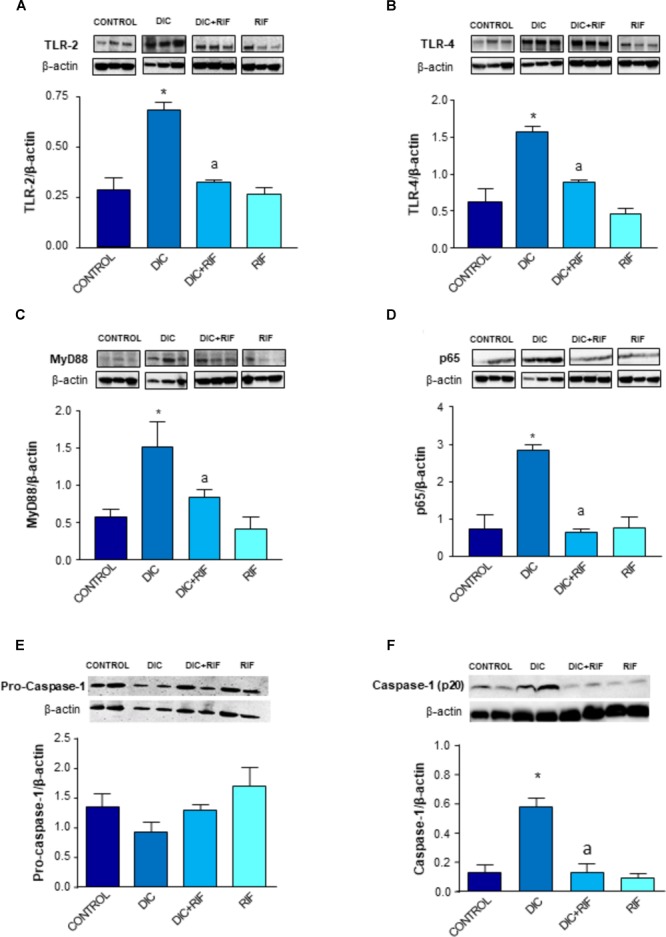
Western blot analysis of toll-like receptor-2 (TLR-2) **(A)**, toll-like receptor-4 (TLR-4) **(B)**, myeloid-differentiation primary response gene 88 (MyD88) **(C)**, activated nuclear factor-κB (p65) **(D)**, pro-caspase-1 **(E)**, and caspase-1 (p20) **(F)** in the ileum of rats treated with vehicle (Control), diclofenac (DIC, 4 mg/kg BID), diclofenac plus rifaximin (DIC+RIF, 50 mg/kg BID), or rifaximin (RIF) for 14 days. Each column represents the mean ± SEM obtained from 10 animals. ^∗^*p* < 0.05, significant difference versus Control; ^a^*p* < 0.05, significant difference versus diclofenac alone. Note that the beta actin signals in **(A,D)** result from stripping and re-probing the same membrane, where TLR-2- and p65 proteins have been detected.

The expression of TLR-2 in small intestinal mucosa was enhanced in animals treated with diclofenac, as compared to controls (Figure 4A). Under these conditions, the co-administration with rifaximin was associated with a significant decrease in the receptor expression, while rifaximin administered alone had no significant effect (Figure 4A).

Rats treated with diclofenac displayed also an increased expression of TLR-4 in ileal mucosa. The administration of diclofenac plus rifaximin elicited a significant reduction of TLR-4 expression (Figure 4B), and again rifaximin alone did not modify it.

Treatment with diclofenac resulted in a significant increment of MyD88 expression in the ileal mucosa (Figure 4C). The administration of rifaximin prevented the diclofenac-induced MyD88 higher expression. In rats treated with rifaximin alone, the levels of MyD88 expression were similar to those detected in control animals (Figure 4C).

The expression of the p65 subunit was increased in the ileum from rats treated with diclofenac, in comparison with control animals (Figure 4D). Under these experimental conditions, co-treatment with rifaximin counterbalanced the higher expression of p65, while rifaximin administration to control animals did not affect the pattern of p65 expression (Figure 4D).

Expression of pro-caspase-1 (50 KD) in ileal tissue did not differ among the experimental groups (Figure 4E). Cleaved caspase-1 (p20, an autoprocessed fragment of caspase-1) expression in ileal tissues from rats treated with diclofenac was significantly increased, in comparison with controls (Figure 4F). The concomitant administration of rifaximin prevented this increase, while rifaximin administered alone did not exert any significant effect (Figure 4F).

### Expression of Occludin

**FIGURE 5:**
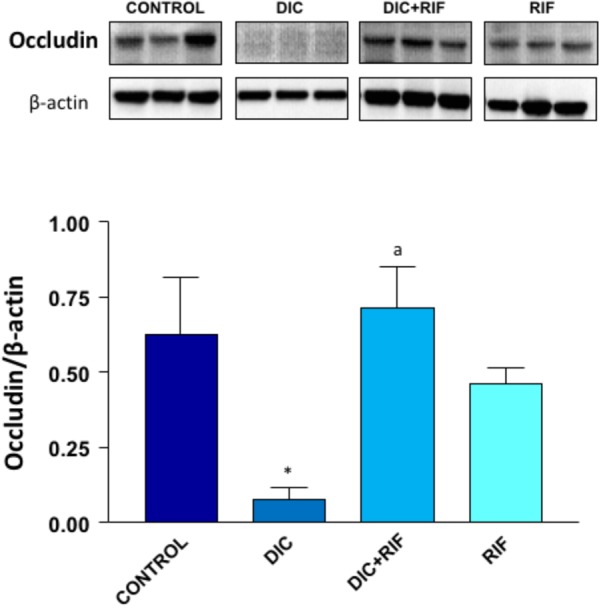
Western blot analysis of occludin in the ileum of rats treated with vehicle (Control), diclofenac (DIC, 4 mg/kg BID), diclofenac plus rifaximin (DIC+RIF, 50 mg/kg BID), or rifaximin (RIF) for 14 days. Each column represents the mean ± SEM obtained from 10 animals. ^∗^*p* < 0.05, significant difference versus Control; ^a^*p* < 0.05, significant difference versus diclofenac alone.

Western blot assay revealed a well-detectable expression of occludin in the ileal mucosa from control animals (Figure 5). By contrast, in rats with diclofenac-induced enteropathy the expression of occludin was markedly reduced. However, the administration of rifaximin to animals with diclofenac-induced enteropathy restored the occludin expression to values similar to those detected in control animals. The administration of rifaximin to normal rats did not significantly change occludin expression (Figure 5).

### Analysis of Bacterial Populations

**FIGURE 6:**
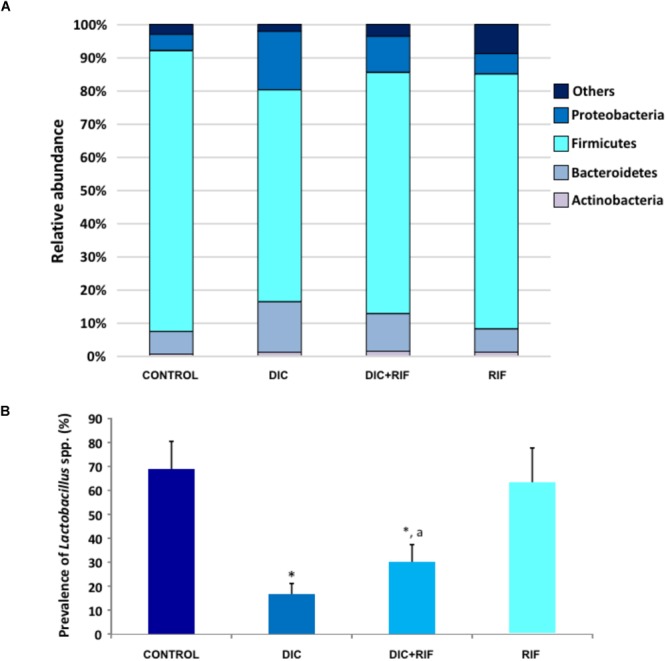
**(A)** Relative abundance of *Proteobacteria, Firmicutes, Bacteroidetes*, and Actinobacteria and **(B)** prevalence of *Lactobacillus* spp. in the ileum of rats treated with vehicle (Control), diclofenac (DIC, 4 mg/kg BID), diclofenac plus rifaximin (DIC+RIF, 50 mg/kg BID), or rifaximin (RIF) for 14 days. Each column represents the mean ± SEM obtained from 10 animals. ^∗^*p* < 0.05, significant difference versus Control; ^a^*p* < 0.05, significant difference versus diclofenac alone.

In control animals, the relative abundance of *Proteobacteria, Firmicutes, Bacteroidetes*, and *Actinobacteria* was 4.8, 84.6, 6.8, and 0.6%, respectively. Treatment with diclofenac was associated with an increase in the relative abundance of *Proteobacteria* and *Bacteroidetes* and a decrease in *Firmicutes* (Figure 6A). The administration of rifaximin to diclofenac-treated rats partially counterbalanced these changes, while rifaximin alone did not exert significant effects (Figure 6A). No relevant changes were observed in the relative abundance of other phyla amongst the different groups (*Data not shown*). When the genus *Lactobacillu*s was specifically analyzed, a substantial decrease was found after diclofenac treatment. Rifaximin partially counterbalanced the NSAID-induced decrease, while it did not affect the relative abundance of *Lactobacillus*, when given alone (Figure 6B). No substantial differences were observed in the prevalence of other main bacterial species among the various groups (*Data not shown*).

### IL-1β Release and Caspase-1 Activation in THP-1 Cells

**FIGURE 7:**
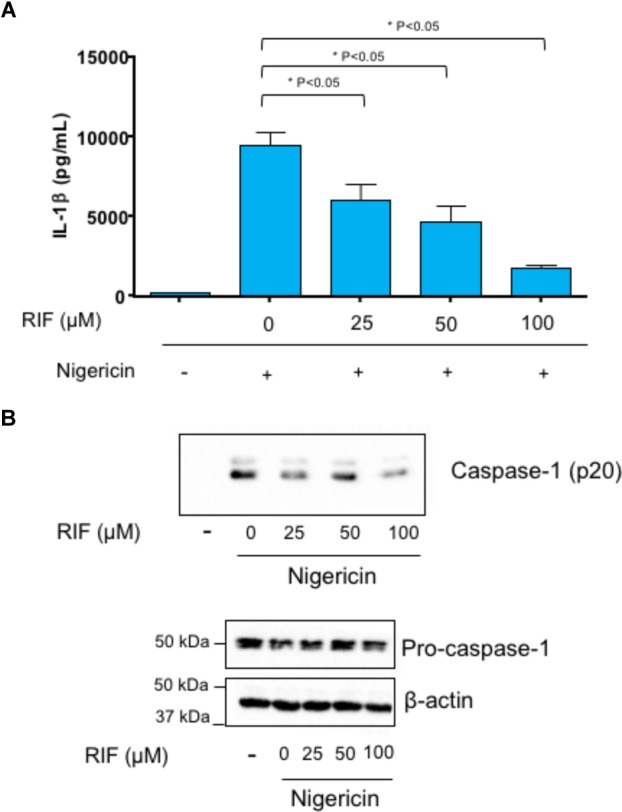
Lipopolysaccharide-primed THP-1 cells treated with rifaximin (RIF, 25, 50, or 100 μM) before the addition of nigericin (10 μM). **(A)** Effect of rifaximin (RIF) on interleukin-1β (IL-1β) release in cell supernatants. **(B)** Effect of rifaximin (RIF) on caspase-1 p20 expression in cell supernatants as well as on pro-caspase-1 expression in cell lysates. Each column represents the mean ± SEM value obtained from four separate experiments. ^∗^*p* < 0.05, significant difference versus cells treated with nigericin alone.

Incubation with nigericin stimulated IL-1β release in lipopolysaccharide-primed THP-1 cells (Figure 7A). Treatment with rifaximin significantly reduced nigericin-induced IL-1β release in a concentration-dependent fashion (Figure 7A).

THP-1 supernatants were assayed for cleavage of caspase-1. Nigericin induced the cleavage of pro-caspase-1 into caspase-1 p20) (Figure 7B). The incubation of THP-1 cells with rifaximin resulted in a decreased activation of caspase-1. By contrast, rifaximin did not affect the expression of pro-caspase-1 in cell lysates (Figure 7B).

## Discussion

Results of the present investigation clearly show that rifaximin administration together with diclofenac protects the small bowel from the damaging effect of this NSAID, in line with the findings obtained in experimental animals with indomethacin (Ciobanu et al., 2014; Fornai et al., 2016) and in healthy volunteers with diclofenac (Scarpignato et al., 2017).

### The Enteropathy Model

The experimental model, set up in our laboratory, was developed to mimic clinical practice with NSAID *chronic* administration in humans. Since elderly patients are more prone to develop enteropathy (Lanas et al., 2012; Watanabe et al., 2013), likely as consequence of an aging gut (Saffrey, 2014) and/or co-morbidities and co-therapies (World Health Organization [WHO], 2011), the present study was conducted on 40-week old rats. Most experimental studies did use single, high doses of NSAIDs (mainly indomethacin) to induce small bowel damage. Under these experimental conditions, the mucosal injury is usually represented by a number of bleeding lesions, affecting deeply the intestinal wall (Abimosleh et al., 2013; Gyenge et al., 2013; Yamamoto et al., 2014). This lesion pattern does not mirror at all the one found during endoscopic investigations in long-term NSAID users (Graham et al., 2005; Maiden et al., 2005; Fujimori et al., 2010). In our experimental conditions, however, diclofenac-induced small bowel damage consisted of diaphragm-like strictures and multiple erosive lesions (along with anemia), similarly to those found in patients receiving long-term NSAID treatment (Maiden et al., 2005).

### Pathophysiology of NSAID-Associated Intestinal Lesions

More than 25 years ago, Bjarnason et al. (1987, 1992) first showed that inflammation and blood loss (evaluated by means of fecal excretion of ^111^In-labeled neutrophils and of ^51^Cr-labeled red cells, respectively) are significantly increased in patients with NSAID-enteropathy, the two parameters being correlated to each other. Both inflammation and blood loss were significantly reduced by 2–12 week treatment with metronidazole (800 mg/day) (Bjarnason et al., 1992). These findings suggested that NSAID-driven intestinal damage is associated with significant mucosal inflammation, which could be easily detected by the increase in fecal calprotectin (Tibble et al., 1999; Maiden et al., 2005; Goldstein et al., 2007), and that the main neutrophil chemo-attractants may be metronidazole-sensitive bacteria.

Along with the intestinal lesions, in our experimental model, inflammation and blood loss paralleled the findings observed in humans. Indeed, MPO, TNF, and IL-1β tissue concentrations as well as fecal calprotectin were significantly increased in rats after diclofenac treatment. In addition, oxidative stress (evaluated as lipid peroxidation, Janero, 1990) also increased in the intestinal mucosa as a consequence of tissue damage. Co-administration of rifaximin with diclofenac counterbalanced all these changes whereas the antibiotic alone did not affect any of these parameters.

Several studies have shown that almost all non-selective NSAIDs induce an early (within 24 h of ingestion) increase of intestinal permeability in humans, an effect that persists after chronic administration (Davies, 1998; Bjarnason and Takeuchi, 2009). Impairment of enteric mucosal barrier represents the *primum movens* of a series of pathophysiologic events, leading to mucosal damage. Indeed, the disrupted intercellular tight-junctions (TJs) facilitate the entrance and action of bacteria or bacterial antigens (as well as other luminal factors), which induce a higher expression and enhanced release of pro-inflammatory cytokines from the intestinal epithelium. Occludin, a 65-kDa tetraspan integral membrane protein, contributes to TJ stabilization and optimal barrier function, and its dysregulation is an established pathophysiologic hallmark of clinical conditions (like NSAID-enteropathy) with underlying alterations of intestinal permeability (Feldman et al., 2005; Cummins, 2012). In this connection, we found a remarkable down-regulation of this TJ protein in the ileal tissue of diclofenac-treated animals. This finding is in line with the decrease in occludin expression induced by indomethacin in Caco-2 (human epithelial colorectal adenocarcinoma) cell monolayers (Carrasco-Pozo et al., 2013).

Disruption of the intestinal TJ barrier is an important pathogenic mechanism leading to increased epithelial penetration of luminal noxious molecules, with consequent activation of the mucosal immune system and subsequent development of intestinal inflammation (Suzuki, 2013). The release of pro-inflammatory cytokines causes further disturbances of the intestinal barrier, thus amplifying immune activation and the inflammatory reaction (Al-Sadi et al., 2009). In our experimental model, together with increased ileal concentrations of TNF and IL-1β, we observed a higher expression of both TLR-4 and TLR-2. In an *acute* model of NSAID-enteropathy, Watanabe et al. (2008) reported up-regulation of TLR-4 suggesting the involvement of Gram-negative bacteria in the pathogenesis of mucosal inflammation and intestinal lesions. Our findings, showing TLR-2 higher expression, suggest a role *also* for Gram-positive microorganisms. Indeed, TLR-4 was found to be a receptor for LPS (major component of the outer membrane of Gram-negative bacteria) while TLR-2 binds peptidoglycan and lipoteichoic acid (major cell wall components of Gram-positive bacteria) (Takeda et al., 2003; McGuire and Arthur, 2015). Stimulation of all TLRs activates MyD88-dependent Nuclear Factor-κB (NF-κB) signaling, which plays a critical role for an effective immune response (McGuire and Arthur, 2015), a pathway observed under our experimental conditions. However, while TLR-2-mediated NF-κB activation is fully MyD88-dependent, LPS activates both MyD88-dependent and independent pathways (Takeuchi and Akira, 2001).

TLR higher expression, along with downstream activation of the NF-κB pathway, suggests the involvement of the NLRP3 inflammasome in the pathogenesis of NSAID-associated intestinal inflammation. Indeed, consistent with this hypothesis, in diclofenac-treated animals, we found an increased cleavage of pro-caspase 1 into caspase-1, followed by elevated ileal concentrations of IL-1β, a well-established pattern of *canonical* NLRP3 inflammasome activation (Pellegrini et al., 2017). In line with these findings, Higashimori et al. (2016) observed – in an *acute* model of indomethacin-induced enteropathy – an increase in mRNA expression of the NLRP3 inflammasome, together with an increase in tissue levels of caspase-1 and IL-1β.

Metagenomic analysis revealed that diclofenac administration induced an increase in the relative abundance of Proteobacteria and Bacteroidetes. Recent investigations reported a marked increase in Proteobacteria in naproxen-treated (Syer et al., 2015) and indomethacin-treated (Fornai et al., 2016) rats, and suggested that these microbial changes might contribute to NSAID-induced intestinal damage. The marked decrease in the *Lactobacillus* genus found in diclofenac-treated animals parallels its almost complete depletion observed in elderly NSAID users (Makivuokko et al., 2010).

In the healthy bowel, commensal non-pathogenic bacteria interact with the host mucosa, co-regulating the function of mucosal barrier. In this context, *Lactobacillus* deficiency may contribute to the development of NSAID-enteropathy. Indeed, these probiotic bacteria exert a protective effect on the intestinal barrier and reduce intestinal permeability (Mennigen and Bruewer, 2009; Liu et al., 2014), and several experimental and human studies (for review see Syer et al., 2015) have shown their ability to prevent NSAID-induced small bowel lesions.

### Mechanisms of Rifaximin Mucosal Protection

The entero-protective effect of rifaximin, observed in this study, was associated with a decrease in tissue inflammation (as reflected by the reduced MPO, TNF, and IL-1β mucosal concentrations) and oxidative stress (mirrored by lowed MDA tissue levels). Along the same lines, diclofenac-induced blood loss and increased fecal calprotectin concentrations were significantly reduced by rifaximin. These findings are in line with the results previously obtained in our laboratories in the setting of indomethacin-enteropathy (Fornai et al., 2016).

New findings in the present investigation concern the reversal by rifaximin of the TLR-2 and TLR-4 higher expression and downstream pro-inflammatory signaling, accompanied by complete recovery of the TJ protein occludin, the expression of which was severely impaired by diclofenac administration. Rifaximin also partially reverted the diclofenac-induced shift of bacterial phyla toward an increase in *Proteobacteria* and *Bacteroidetes* abundance. Most importantly, the drug also increased the proportion of Lactobacilli, a genus depleted by the NSAID.

The nature of the anti-inflammatory activity of rifaximin could be the consequence of its anti-microbial properties (and therefore indirect) or intrinsic to the pharmacologic properties of the drug (i.e., a direct one). Drugs belonging to the rifamycin class exert anti-inflammatory activity, which seems to be independent from the anti-microbial properties. Rifamycins indeed inhibit human neutrophil functions (Spisani et al., 1997, 1998) and intra-articular rifamycin SV was shown to be effective in patients with chronic arthritides, like juvenile rheumatoid arthritis and ankylosing spondylitis (Caruso, 1997).

Some studies, including our own, have shown the rifaximin ability of inhibiting inflammatory cytokine release in the intestinal mucosa, induced by chronic stress (Xu et al., 2014) or NSAID administration (Fornai et al., 2016) in rats. In addition, the drug reduced cytokine production from LPS-activated THP-1 monocytes *in vitro* (Rosette et al., 2013), thus suggesting an *intrinsic* anti-inflammatory activity. This last investigation, however, did not provide any mechanistic explanation for the anti-inflammatory effect of rifaximin. Since we (this study) and others (Higashimori et al., 2016) have shown that NSAID-associated intestinal mucosal inflammation is mediated via NLRP3 inflammasome activation, an effect counterbalanced by rifaximin administration, we hypothesized that the anti-inflammatory activity of this antibiotic could rely on a modulation of NLRP3 inflammasome activity. Therefore, rifaximin was tested in an *in vitro* cell system (LPS-primed THP-1 cells stimulated by nigericin) for its ability to modulate NLRP3 inflammasome pathway. In accordance with our hypothesis, the drug reduced IL-1β production in a dose-dependent fashion, this effect being associated with inhibition of the up-stream caspase-1 activation. This original observation provides compelling evidence for an *intrinsic* anti-inflammatory activity of rifaximin.

### Rifaximin for Prevention of NSAID-Enteropathy in Clinical Practice

Recent evidence is consistent with the idea that – besides non-anti-microbial activities (e.g., anti-inflammatory action) – rifaximin displays also “eubiotic” properties. In patients with inflammatory conditions of the digestive system (inflammatory bowel disease, colonic diverticular disease or hepatic encephalopathy), this antibiotic – conversely from systemic anti-microbials – did not change the overall human colonic microbiota, but actually increased the relative abundance of Bifidobacteria and Lactobacilli (Maccaferri et al., 2010; Xu et al., 2014; Ponziani et al., 2016).

NSAID therapy is often required in long-term, especially in chronic inflammatory conditions. As a consequence, also prevention of NSAID gastrointestinal injury needs to be long-term and should be accomplished with effective and safe drugs. Rifaximin proved to be extremely safe. Indeed, its minimal, if any, systemic absorption translates into an adverse event profile, overlapping that of placebo, even after 6-month continuous administration (Bass et al., 2010). In addition to the lack of emergence of pathogenic bacteria, occurrence of opportunistic infections, and alteration of the overall microbiota, no clinically relevant changes in bacterial sensitivity to other antibiotic classes have been reported (DuPont et al., 2016; Pimentel et al., 2017).

*In summary*, co-administration of rifaximin with diclofenac prevents NSAID-induced small bowel damage in rats. These results and those of a recent proof-of-concept study in humans (Scarpignato et al., 2017) suggest that targeting enteric bacteria and mucosal inflammation by a poorly absorbed antibiotic, such as rifaximin, is an attractive therapeutic avenue for the prevention of NSAID-enteropathy (Scarpignato, 2008).

## Author Contributions

CB and CS had full access to all of the data in the study and take the responsibility for the integrity of the data, study concept, obtained funding, and drafting of the manuscript. CS, RC, GL-C, MF, and CR: study design. RC, CP, MF, EP, ET, DG, LB, FF, PP-R, CS, and CB: collection, analysis, and interpretation of data. RC, EG, GN, CB, and CS: critical revision of the manuscript for important intellectual content. MF and LA: statistical analysis.

## Conflict of Interest Statement

CS is member of the Speakers’ Bureau and of the Scientific Advisory Board of Alfasigma SpA, the manufacturer of rifaximin. CB is member of the Speakers’ Bureau of Alfasigma SpA. The remaining authors declare that the research was conducted in the absence of any commercial or financial relationships that could be construed as a potential conflict of interest.
