# Supplementary material for: Pathophysiology of NSAID-Associated Intestinal Lesions in the Rat: Luminal Bacteria and Mucosal Inflammation as Targets for Prevention
Source: Front Pharmacol. 2018 Nov 29;9:1340. doi: 10.3389/fphar.2018.01340 (PMC6281992; doi:10.3389/fphar.2018.01340)
Supplement: Supplementary file 1 [file Table_1.docx]

**Supplementary Table 1.** Microscopic criteria for quantitative estimation of the intestinal injury elicited by diclofenac

| **Type 1 injury** | - Damage confined to the tunica mucosa - De-epithelization - Significant morphologic alterations of villi |
| --- | --- |
| **Type 2 injury** | - Inflammatory infiltration in the submucosa, with thickening of the tunica muscularis or serosa - The morphologic framework of tunica mucosa is preserved |
| **Type 3 injury** | - Damage involves the full thickness of intestinal wall - The morphologic patterns of tunicae are lost - Inflammatory reaction widely extended to the tunica serosa with a significant increase in thickness |

**Supplementary Table 2.** Primary and secondary antibodies employed in the western blot assays

| Primary antibody | Code# | Host/Type | Vendor | Dilution |
| --- | --- | --- | --- | --- |
| TLR-2 | Sc-10739 | Rabbit polyclonal | Santa Cruz Biotechnology | 1.1000 |
| TLR-4 | Ab22048 | Mouse monoclonal | Abcam | 1:1000 |
| MyD88 | Sc-11356 | Rabbit polyclonal | Santa Cruz Biotechnology | 1:1000 |
| p65 (NF-kB) | Sc-372 | Rabbit polyclonal | Santa Cruz Biotechnology | 1:1000 |
| Pro-Caspase 1 | ab108362 | Rabbit monoclonal | Abcam | 1:1000 |
| Caspase 1 p20 | Sc-514 | Rabbit polyclonal | Santa Cruz Biotechnology | 1:1000 |
| Occludin | Sc-5562 | Rabbit polyclonal | Santa Cruz Biotechnology | 1:1000 |
| β-actin | 3854 | Mouse monoclonal | Sigma | 1:5000 |
| Caspase-1 p20 | 3866 | Rabbit monoclonal | Cell Signaling Technology | 1:200 |
| Pro-Caspase 1 | Sc-515 | Rabbit polyclonal | Santa Cruz Biotechnology | 1:1000 |
|  |  |  |  |  |
| Secondary antibody |  |  |  |  |
| Anti-rabbit IgG-HRP | Sc-2004 | Goat polyclonal | Santa Cruz Biotechnology | 1:10000 |
| Anti-mouse IgG-HRP | Sc-2005 | Goat polyclonal | Santa Cruz Biotechnology | 1:10000 |
| Anti-rabbit IgG-HRP | P0448 | Goat polyclonal | Dako | 1:2000 |
