# Supplementary material for: Pathophysiology of NSAID-Associated Intestinal Lesions in the Rat: Luminal Bacteria and Mucosal Inflammation as Targets for Prevention
Source: Front Pharmacol. 2018 Nov 29;9:1340. doi: 10.3389/fphar.2018.01340 (PMC6281992; doi:10.3389/fphar.2018.01340)
Supplement: Supplementary file 2 [file Data_Sheet_1.docx]

**Supplementary Material**

**Detailed Methodology Used in the Study**

1**.** Microscopic Assessment of Intestinal Damage

Histological evaluation of small bowel injury was carried out as previously described[^1^](#_ENREF_1) . Upon removal, the small intestine was immediately injected with 10 % formalin and left in the same fixative solution. After 30 minutes, it was opened along the anti-mesenteric border, cleaned of its fecal contents, and fixed again in 10 % formalin for 24 hours. In order to rule out any bias, ileal samples were taken in accordance to the following procedure: the full length of small intestine was measured; 1 cm of tissue proximal to the ileo-cecal valve was discarded, and two specimens of 1.5-2 cm were taken at this edge as well as 5 cm from the first ileum sample[^2^](#_ENREF_2).

Sections of ileum were embedded into paraffin blocks and cut into 3 consecutive serial 7-8 µm sections. The slices were cut at two different points of the block: two on the surface and three at a deeper level. Each slice was placed on a glass slide for staining with haematoxylin and eosin. Histological damage was assessed by two observers, blind to treatments, according to the score system adopted in our laboratory[^1^](#_ENREF_1)^,2^. The intestinal damage was scored as reported in Supplementary Table 1. Representative pictures, showing the histological appearance of normal ileum, as well as type 1, 2 and 3 lesions are displayed in Figure 1A, B, C and D, respectively.

2. Evaluation of Tissue Myeloperoxidase Levels

MPO, as a quantitative index to estimate the degree of intestinal wall infiltration by inflammatory polymorphonuclear cells, was assessed as described by Fornai et al.[^1^](#_ENREF_1). Specimens of small intestinal tissues (30 mg) were homogenized on ice with a polytron homogenizer (QIAGEN, Milan, Italy) in 0.6 mL of ice-cold lysis buffer (200 mM NaCl, 5 mM EDTA, 10 mM Tris, 10% glycerine, 1 mM phenylmethylsulfonyl fluoride, 1 μg/ml leupeptin and 28 μg/ml aprotinin, pH 7.4). The homogenate was centrifuged 2 times at 4°C for 15 minutes at 1,500 *g*. The supernatant was diluted 1:5 and used for determination of MPO concentration by means of an enzyme-linked immunosorbent assay (ELISA; Hycult Biotech, Uden, The Netherlands). The results were expressed as nanograms of MPO per milligram of intestinal tissue.

3. Evaluation of Tissue Malondialdehyde Levels

MDA concentration in intestinal tissues was determined to obtain quantitative estimates of membrane lipid peroxidation[^2^](#_ENREF_2). For this purpose, intestinal tissue was excised, weighed, minced by forceps, homogenized in 2 ml of cold buffer (Tris-HCl 20 mM, pH 7.4) using a polytron homogenizer (QIAGEN, Milan, Italy), and centrifuged at 1,500 *g* for 10 minutes at 4°C. Aliquots of supernatants were then used for subsequent assay procedures. Mucosal MDA concentrations were estimated using a colorimetric assay kit (Cayman Chemical, Ann Arbor, MI, U.S.A.). Results were expressed as nanomoles of MDA per milligram of intestinal tissue.

4. Evaluation of Tissue TNF Levels

Tissue levels of TNF, a potent inflammatory cytokine, whose intestinal production is increased dose-dependently by NSAIDs, like indomethacin[^3^](#_ENREF_3), were measured as previously described[^4^](#_ENREF_4). Briefly, samples of ileum, collected as reported above, were weighed, thawed, and homogenized in 0.4 ml of phosphate buffered saline (0.4 ml/20 mg of tissue), pH 7.2 at 4°C, and centrifuged at 10,000 *g* for 5 minutes. Aliquots (100 μL) of the supernatants were then used for subsequent assay by means of a commercial ELISA kit (Abcam, Cambridge, UK). Tissue TNF levels were expressed as picograms per milligram of tissue.

5. Evaluation of Tissue IL-1β Levels

IL-1β levels in ileal tissues were measured by enzyme-linked immunosorbent assay kits (Abcam, Cambridge, UK), as previously described[^5^](#_ENREF_5). Briefly, samples of ileum, collected as reported above, were weighed, thawed, and homogenized in 0.4 ml of phosphate buffered saline (0.4 ml/20 mg of tissue), pH 7.2 at 4°C, and centrifuged at 10,000 *g* for 5 minutes. Aliquots (100 μL) of supernatants were then used for assay. Tissue IL-1β levels were expressed as picograms per milligram of tissue.

6. Assay of Fecal Calprotectin

Fecal pellets, collected as reported above, were frozen dried for 24 hours and reconstituted in 1 mL PBS, along with 50 μL 1% (wt/vol) ascorbic acid (Sigma, St Louis, MO, USA). Samples were then homogenized for 10 minutes (4°C). Homogenates were diluted with 2 mL lysis buffer (0.1 % sodium dodecylsulfate, 0.5 % sodium deoxycholate, 0.02 % sodium azide, 5 mM disodium ethylenediaminetetraacetic acid, and 1× Halt protease/phosphatase inhibitor cocktail (Thermo Fisher Scientific Inc, Waltham, MA, USA) in PBS. Homogenates were further homogenized for 30 seconds and centrifuged (5,800 *g*, 10 minutes, 4°C), and supernatants were snap frozen in liquid nitrogen and stored at −80°C. Fecal calprotectin levels were determined using a specific rat calprotectin enzyme-linked immunosorbent assay kit (DBA, Milan, Italy). Homogenates were diluted 2-fold with phosphate buffered saline and analyzed following the manufacturer's instructions. Results were expressed as micrograms of calprotectin per gram of feces.

7. Assessment of Blood Hemoglobin Concentration

Haemoglobin analysis was performed on blood samples, collected as reported above, by means of Quantichrom Hemoglobin assay kit (Bioassay Systems, Hayward, CA, USA) and expressed as g/dL.

8. Western Blot Analysis of TLR-2, TLR-4, MyD88, NF-kB p65 Subunit, Caspase-1 and Occludin

Specimens of mucosa were excised from ileum, weighed and homogenized in lysis buffer containing: HEPES 10 mmol/L, NaCl 30 mmol/L, ethylenediaminetetraacetic acid 0.2 mmol/L, phenylmethylsulfonyl fluoride 2 mmol/L, leupeptin 10 µg/mL, aprotinin 10 µg/ml, sodium fluoride 1 mmol/L, sodium orthovanadate 1 mmol/L, glycerol 2%, MgCl_2_ 0.3 mmol/L, and Triton-X 100 1 %, using a polytron homogenizer. Mucosal homogenates were spun by centrifugation at 15,000 *g* for 15 minutes at 4°C, and the resulting supernatants were then separated from pellets and stored at −80°C. Protein concentration in each sample was determined by the Bradford method[^6^](#_ENREF_6) (Protein Assay Kit, Bio-Rad, Hercules, CA, USA). To perform western blot analysis of TLR-2, TLR-4, MyD88, p65 (the subunit resulting from NF-kB activation[^7^](#_ENREF_7)), caspase-1 and occludin, equivalent amounts of protein lysates (50 µg) were separated by electrophoresis. The blots were then blocked overnight with 5 % non-fat dried milk in phosphate buffered saline, and incubated overnight at room temperature with primary antibodies (Supplementary table 2). After repeated washings with 0.1 % Tween-20 in Tris-buffered saline, horseradish peroxidase-conjugated secondary antibodies (Supplementary table 2) were added for 1 hour at room temperature. After repeated washings with 0.1 % Tween-20 in Tris-buffered saline, immunoreactive bands were visualized by incubation with chemiluminescent reagents and exposed to Kodak Image Station 440 for signal detection and densitometric image analysis. To ensure equal sample loading, blots were stripped and reprobed for determination of β-actin by a specific primary antibody (Supplementary Table 2).

9. Analysis of Bacterial Populations in Ileal Samples

9.1 *DNA Extraction*

Ileal tissue samples were stored at -80°C immediately after collection. DNA was extracted from 25 mg of ileal tissue using the QIAamp DNA Mini kit (Qiagen, Hildens, Germany) and following the manufacturer’s instructions. The final elution volume was 200 µl and the DNA concentration was determined by absorbance at 260 nm (A260), and the purity was estimated by determining the A260/A280 ratio with a spectrophotometer.

9.2 *Metagenomic Analysis (16 S RNA Gene Amplification and MiSeq Sequencing)*

Total DNA extracted from ileal tissues was analyzed for the mucosal-associated microbiota through 16S rDNA metagenomics. Metagenomic analysis was performed with MiSeq, Illumina platform at GenProbio SRL (Parma, Italy). Briefly, amplicons of the V3 bacterial 16S rRNA gene region were obtained using primer pair Probio_Uni and Probio_Rev, as previously reported[^8^](#_ENREF_8). The 16S rRNA gene amplicons were prepared following the 16S Metagenomic Sequencing Library Preparation Protocol. PCR products were purified by magnetic purification step and DNA concentrations were measured using fluorimetric Qubit quantification system. Amplicons were diluted to appropriate concentration and mixed to prepare the pooled final library (2). Sequencing was performed using the Illumina MiSeq platform, with MiSeq Reagent Kit v3 chemicals. The number of sequences and operational taxonomic units (OTUs) for each sample were calculated using Quantitative Insights Into Microbial Ecology software (QIIME, <http://qiime.org/scripts/version1.9.1>) and taxonomic classification on Silva v. 119 database was assessed[^9^](#_ENREF_9)^,^ [^10^](#_ENREF_10)

10. *In Vitro* Assays of NLRP3 Inflammasome

10.1 *Measurement of IL-1****β*** *Concentrations*

Cell supernatants were analyzed for IL-1β using specific ELISA kits (R&D Systems, catalog number DY201) according to the manufacturer’s instructions.

10.2 *Western Blot Assay of Caspase-1*

Cells were lysed for 20 minutes on ice using lysis buffer (150 mM NaCl; 50 mM Tris-HCl pH 8; 10 % Glycerol; 1 mM ethylenediaminetetraacetic acid; 1 % Triton), supplemented with protease inhibitors (Sigma, P8340), and the insoluble fraction was removed by centrifugation (12,500 *g*, 10 minutes). Cell supernatants were concentrated using Amicon 10k columns (Merck, FC501096). For the detection of pro-caspase-1, activated caspase-1 and β-actin, cell lysates (30 µg) and supernatants were resolved on 12 % polyacrylamide gels. Proteins were transferred to a nitrocellulose membrane, and specific proteins were detected by Western blotting with primary antibodies (Supplementary Table 2), followed by a secondary horseradish peroxidase-conjugated antibody (Supplementary Table 2), and subsequently detected using enhanced chemiluminescence reagents (ECL, Amersham Biosciences).

.

**References**

1. Fornai M, Antonioli L, Colucci R, et al. NSAID-induced enteropathy: are the currently available selective COX-2 inhibitors all the same? J Pharmacol Exp Ther 2014;348:86-95.

2. Fornai M, Antonioli L, Pellegrini C, et al. Small bowel protection against NSAID-injury in rats: Effect of rifaximin, a poorly absorbed, GI targeted, antibiotic. Pharmacol Res 2016;104:186-96.

3. Bertrand V, Guimbaud R, Tulliez M, et al. Increase in tumor necrosis factor-alpha production linked to the toxicity of indomethacin for the rat small intestine. Br J Pharmacol 1998;124:1385-94.

4. Antonioli L, Fornai M, Colucci R, et al. Inhibition of adenosine deaminase attenuates inflammation in experimental colitis. J Pharmacol Exp Ther 2007;322:435-42.

5. Pellegrini C, Fornai M, Colucci R, et al. Alteration of colonic excitatory tachykininergic motility and enteric inflammation following dopaminergic nigrostriatal neurodegeneration. J Neuroinflammation 2016;13:146.

6. Bradford MM. A rapid and sensitive method for the quantitation of microgram quantities of protein utilizing the principle of protein-dye binding. Anal Biochem 1976;72:248-54.

7. Oeckinghaus A, Ghosh S. The NF-kappaB family of transcription factors and its regulation. Cold Spring Harb Perspect Biol 2009;1:a000034.

8. Milani C, Hevia A, Foroni E, et al. Assessing the fecal microbiota: an optimized ion torrent 16S rRNA gene-based analysis protocol. PLoS One 2013;8:e68739.

9. Milani C, Ticinesi A, Gerritsen J, et al. Gut microbiota composition and Clostridium difficile infection in hospitalized elderly individuals: a metagenomic study. Sci Rep 2016;6:25945.

10. Mancabelli L, Ferrario C, Milani C, et al. Insights into the biodiversity of the gut microbiota of broiler chickens. Environ Microbiol 2016;18:4727-4738.
