# Supplementary figures and images for: Pathophysiology of NSAID-Associated Intestinal Lesions in the Rat: Luminal Bacteria and Mucosal Inflammation as Targets for Prevention
Source: Front Pharmacol. 2018 Nov 29;9:1340. doi: 10.3389/fphar.2018.01340 (PMC6281992; doi:10.3389/fphar.2018.01340)

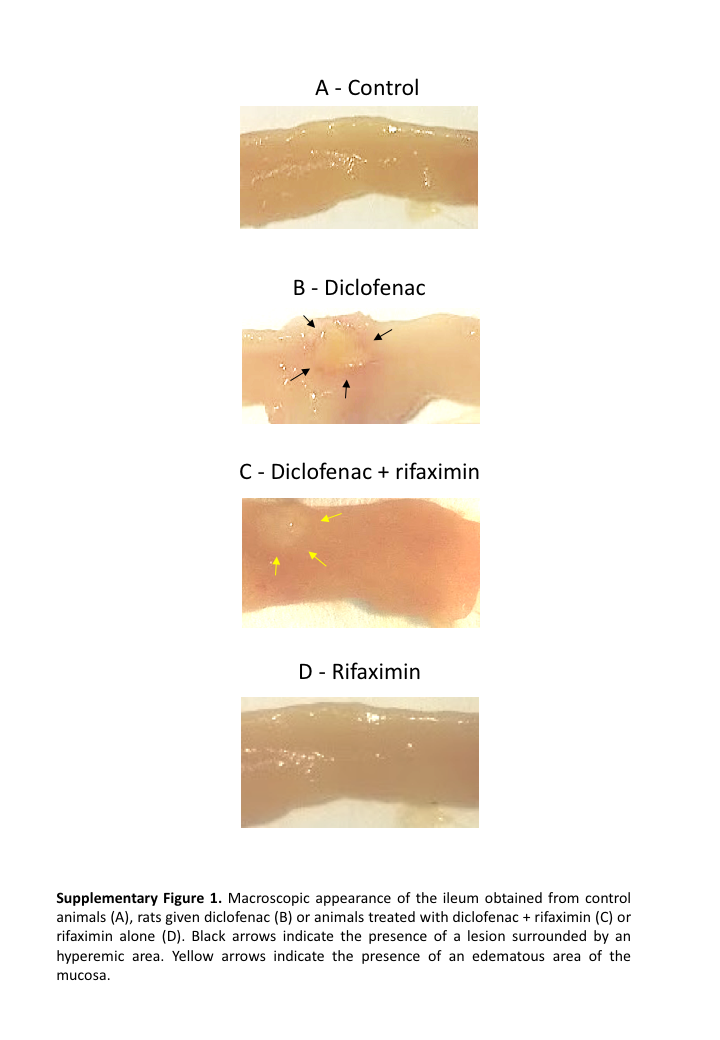

Supplement: Supplementary file 3 [file Image_1.TIFF]
